# Massive single visit cervical pre-cancer and cancer screening in eastern Democratic Republic of Congo

**DOI:** 10.1186/s12905-019-0737-y

**Published:** 2019-03-04

**Authors:** Justin Lussy Paluku, Tamar E. Carter, Miriam Lee, Susan A Bartels

**Affiliations:** 1 HEAL Africa Hospital, Goma, Democratic Republic of Congo; 2 Bioinformatics and Genomics Department, University of North Carolina, Charlotte, USA; 3 Department of Biology, Baylor University, Waco, TX USA; 4 Pindara Hospital, Queensland, Australia; 5 Departments of Emergency Medicine and Public Health Sciences, Queen’s University, Kingston, Canada

**Keywords:** Cervical cancer, Democratic Republic of Congo, Screening, Loop electrosurgical excision procedure, Screen and treat, VIA, VILI

## Abstract

**Background:**

In the Democratic Republic of Congo (DRC), practical and affordable strategies for cervical cancer screening are needed to detect and treat pre-cancerous and cancerous lesions in a timely fashion. This study presents the results of mass cervical cancer screenings in eastern DRC using a “screen and treat” approach.

**Methods:**

In two mass cervical cancer screening campaigns, patients underwent a combination of visual inspection of the cervix with acetic acid, visual inspection of the cervix with Lugol iodine solution, and colposcopy with or without loop electrosurgical excision procedure. Cervical biopsy samples were taken for histology analysis. Marital status, age, history of abnormal bleeding, and number of pregnancies were recorded for each patient and association analyses were performed.

**Results:**

Of the 644 women who received cervical pre-cancer and cancer screening, 48 had suspicious pre-cancer and cancer lesions that were biopsied (7.45%). On histology analysis cervical intraepithelial neoplasia (CIN) was identified in 15 (2.33%), squamous cell carcinoma (SCC) was identified in 6 (0.93%) and non-neoplastic cervicitis was identified in 11 (1.71%). Abnormal bleeding was significantly associated with CIN/SCC but no significant association was observed for prior pregnancy, patients’ home region, or age.

**Conclusion:**

Forty-eight women with suspicious pre-cancerous or cancerous lesions were successfully identified using the “screen and treat” approach in eastern DRC, suggesting that this approach is feasible for reducing cervical cancer morbidity and mortality. However, community awareness would be necessary, providers would have to be properly trained, referral and follow up mechanisms would have to be put in place, and equipment / supplies would have to be secured if the “screen and treat” approach is to be successful on a wider scale. There is ongoing need for HPV vaccination in DRC as a primary prevention strategy against cervical cancer.

## Background

Cervical cancer is the second most common cancer among women globally, with an estimated 528,000 new cases and 266,000 deaths annually worldwide [1]. Women in low-income countries are disproportionately affected and in 2012, 85% of all new diagnoses of cervical cancer as well as 87% of all cervical cancer deaths were reported from low and middle- income countries (LMIC) [1]. While cervical cancer rates vary across LMIC, some of the highest rates have been reported from Eastern Africa [2]. Well-organized screening and treatment programs for pre-cancerous and cancerous lesions as well as introduction of the human papillomavirus (HPV) vaccination have contributed to the reduced incidence of cervical cancer in developed countries [3–5]. However, in low-income countries cervical cancer screening and treatment programs are few in number and often limited in capacity. As a result, low-income countries have not had the same reduction in cervical cancer incidence as higher income countries [1]. In fact, cervical cancer remains the second most common cancer affecting women in developing countries (17.8 per 100,000 women [4]) and in the continent of Africa, cervical cancer is the leading cause of cancer-related deaths among women [5].

Cervical cancer is a preventable and treatable disease. Related morbidity and mortality could be reduced in LMIC by efficient screening and prompt treatment of pre-cancerous and cancerous lesions [6]. This “screen and treat” strategy is aimed at the identification of suspicious cervical lesions using a combination of visual methods [visual inspection of the cervix with acetic acid (VIA) or visual inspection of the cervix with Lugol iodine solution (VILI)], colposcopy and immediate treatment with cryotherapy or loop electrosurgical excision procedure (LEEP) [7]. Such a program is less expensive and easier to implement than the screening programs that exist in most developed countries. The “screen and treat” approach has the potential to reduce the burden of cervical cancer in resource-poor settings [3].

The Democratic Republic of Congo (DRC) is a resource-limited country ranking 176 out of 188 on the human development index [8]. In DRC, the health system is poorly organized and under-funded, and many governmental and mission hospitals have been destroyed by decades of armed conflict. Because the surviving hospitals are often poorly equipped and most lack qualified medical personnel, women in DRC have little or no access to preventive and curative services such cervical cancer screening and treatment. There is currently very limited literature on cervical cancer screening in the DRC [9].

To address this need, a pilot cervical cancer screening program, utilizing the “screen and treat” approach, was initiated by the HEAL Africa Hospital. HEAL Africa is a full-service hospital in Goma, the capital city of North Kivu Province in DRC, which has an estimated population of approximately 1.5 million. It offers inpatient and outpatient services including internal medicine, general and orthopedic surgery, obstetrics and gynecology, as well as radiology. At the time of the mass cervical cancer screenings, two gynecologists and two family medicine doctors at HEAL Africa were available to participate in the screening program. The project’s research objectives were: a) to evaluate the feasibility of a screen and treat approach for cervical cancer in this context and b) to determine the prevalence of CIN/SCC in this patient population. These results are reported here along with the demographic and clinical characteristics of patients screened.

## Methods

### Study setting and patients

This cervical cancer screening pilot was conducted in eastern DRC at the HEAL Africa Hospital in Goma, North Kivu Province. Women were informed about the program through local radio announcements and local churches using recruitment campaigns distributed in French and Swahili. The recruitment message was culturally appropriate and included the following information: a) known risk factors for cervical cancer include early maternity, multiple sex partners and poverty; b) abnormal vaginal bleeding can be a symptom of cervical cancer; c) cervical cancer can be fatal; and d) free screening and treatment available at HEAL Africa Hospital at the designated dates. The message was delivered at church services and on local radio programming for two weeks prior to each of the screening programs. Interested women aged 20 to 65 were requested to report to the hospital and be registered for cervical cancer screening during pre-defined screening periods. The first mass screening was conducted from August 21 to 24, 2013 and the second from August 3 to August 7, 2015.

### Screening method

A single team conducted both screening programs and was composed of: 1) a gynecologist / accredited colposcopist from Australia with twenty years experience in cervical cancer screening, 2) two Congolese gynecologists, and 3) two Congolese nurses. Before the first screening campaign, the local physicians and nurses participated in a half-day training session led by the visiting expert from Australia. At the end of the half-day training, local staff were comfortable conducting the LEEP procedure but guidance and support was provided by the visited expert throughout the two screening campaigns. Screening colposcopy and ‘screen and treat’ methods were adopted due to the lack of cytology services or HPV testing in Goma. Gynecologic examinations were conducted by the gynecologists.

#### Visual inspection of the cervix with acetic acid (VIA) and colposcopy

For each patient, a speculum was inserted in the vagina to visualize the cervix. Acetic acid, 5%, was applied to the cervix and after waiting for approximately 60 s, the cervix was inspected using a bi-locular colposcope for any aceto-white changes indicating a pre-cancerous lesion. Absence of aceto-white lesions was categorized as normal.

#### *Visual inspection of the cervix with Lugol iodine solution* (VILI) *and colposcopy*

Lugol’s iodine is more sensitive in detecting dysplastic changes and was therefore used to further confirm aceto-white changes. The Lugol iodine solution was applied to any cervix identified as having aceto-white lesions and the cervix was inspected using the same colposcope. Results of VILI and colposcopy were categorized as follows:

1. Aceto-white lesions that did not take up the Lugol’s solution - low or high-grade cervical intraepithelial neoplasia (CIN);
2. Light aceto-white lesions either abutting or not abutting the squamocolumnar junction - low-grade CIN;
3. Dense aceto-white lesions or mosaicism abutting or not abutting the squamocolumnar junction - high-grade CIN; and
4. Friable masses with irregular surfaces that bled easily on contact - suspicious for squamous cell carcinoma (SCC).

#### Colposcopy-directed treatment and biopsy sampling

Any cervical lesion identified as suspicious for CIN or SCC on colposcopic exam after application of acetic-acid 5% and Lugol’s solution, was removed using LEEP. After informed consent had been given, LEEP was performed using universal aseptic precautions with the patient in lithotomy position and using a Cuscos speculum. Circumferential cervical block was performed using Lignospan Special (Lignocaine hydrochloride 2% and adrenaline tartrate 1/80.000) with a Terumo Dental Needle®. Loop electrodes of varying sizes were used depending on the size of the cervical lesion (20 × 8 mm, 15 × 12 mm, 15 × 6 mm, 10 × 10 mm). Coagulation was applied to the base of the lesion and to the edges of the cervix after excision. Any lesion suspected to be cancerous was biopsied with a TISCHLER Cervical Biopsy Punches Forceps. Punch biopsies and LEEP specimens were placed in 10% formalin and sent to Kampala, Uganda for histopathologic examination by a consultant. Patients were followed up after the LEEP / punch biopsies and results were given to them on a subsequent visit.

### Statistical analysis

Demographic data and a relevant medical history were collected from patients by one of the Congolese gynecologists or nurses. Collected data included residential address, age, parity, marital status, last normal menstrual period, and history of abnormal or post-coital bleeding in the previous year, as well as past medical and surgical history. Data regarding education level, number of sexual partners and age of first sexual intercourse was not collected due to time constraints. Descriptive statistics for demographic variables (marital status, age, region of residence) and relevant clinical characteristics (history of abnormal bleeding in the previous year, prior pregnancies) of all patients screened were generated. For association analyses, region of residence was treated as a categorical variable and age was treated as a continuous variable. Marital status (married versus not married), history of prior pregnancy (> 1 versus none) and history of abnormal vaginal bleeding in the year prior (yes versus no) were all treated as dichotomous variables. The outcome of screening / biopsy results was also treated as a dichotomous variable (normal versus CIN/SCC). Bivariate analysis to test the association of patient demographic and clinical characteristics with cervical screening / biopsy results was performed. Fisher Exact Test was used to examine the association between screening/ biopsy results and marital status, abnormal vaginal bleeding, and prior pregnancy. Chi-square analysis was used to examine association between screening / biopsy results and patients’ region of residence. Logistic regression was used to test the association between screening/ biopsy results and patients’ age. An alpha level of 0.05 was used for all statistical tests. Analysis were performed using SAS 9.4 (SAS Enterprise, Cary, NC, USA).

## Results

In the first cervical cancer screening campaign (August 21–24, 2013), 233 patients were screened and in the second (August 3–7, 2015), 411 patients were screened giving a total of 644 patients. Patient medical records were retained at HEAL Africa Hospital and no individual woman was screened twice. Demographic characteristics are summarized in Table 1. The majority of participants were married (77.80%) and over the age of 30 (80.59%; mean age = 38.78, standard deviation (SD) = 10.9). A majority of the patients did not report a history of abnormal bleeding (60.25%) and had had one or more pregnancies (90.37%). All screened patients reported that they were sexually active.

**Table 1:** Demographic and clinical characteristics of patients participating for a mass cervical cancer screening and treatment program at HEAL Africa Hospital (*N* = 644)

	Frequency	Percent
Age		
< 20	2	0.31
20–29	123	19.10
30–39	261	40.53
40–49	150	23.29
50–59	108	16.77
Marital Status		
Married	501	77.80
Widowed	60	9.62
Single (never married)	53	8.23
Divorced	27	4.19
Separated	3	0.47
Region of Residence		
Outside Goma	204	31.68
Keshero and Himbi	166	25.78
Mabanga and Virunga	164	25.47
Birere	65	10.09
Ville	44	6.83
No response	1	0.16
Obstetrical History		
> 1 pregnancy	582	90.37
No prior pregnancies	61	9.47
No response	1	0.16
History of Abnormal Vaginal Bleeding in Prior Year		
No	388	60.25
Yes	256	39.75

Keshero, Himbi, Mabanga, Virunga, Birere, and Ville are all neighborhoods in the city of Goma

Table 2 summarizes the results of the cervical cancer screening and biopsies. Of the 644 women screened, 48 (7.45%) had biopsy samples taken from suspicious lesions identified on VIA and VILI colposcopic exam. Of these 48 patients, 70.83% were married, 83.33% were over the age of 30 (mean age = 38.5), 87.50% reported abnormal bleeding, and 89.58% had had one or more pregnancies. On histological analysis, CIN was identified in 15 patients (31.24% of biopsied samples) and SCC in 6 patients (12.50% of biopsied samples). Cervicitis without CIN or SCC was identified in 11 women (22.92% of biopsied samples) and one case of tuberculosis was diagnosed. Eleven biopsies were inconclusive (22.92%). Of the 644 women screened, CIN and SCC had prevalences of 2.34 and 0.93%, respectively. Three patients who underwent a LEEP experienced minor vaginal bleeding. There were no post-procedure infectious complications and no major bleeding.

**Table 2:** Results of VIA / VILI / colposcopy cervical cancer screening and biopsy findings

	Frequency	Percentage of total screened	Percentage of total biopsied
Normal screen	596	92.54	–
Biopsy Results	48	7.46	100
Normal	4	0.62	8.33
CIN1	3	0.47	6.25
CIN1 and cervicitis	4	0.62	8.33
CIN2	3	0.47	6.25
CIN2 and cervicitis	1	0.16	2.08
CIN3	4	0.62	8.33
Squamous cell carcinoma	6	0.93	12.50
Non-neoplastic cervicitis	11	1.71	22.92
Tuberculosis	1	0.16	2.08
Inconclusive	11	1.71	22.92

Table 3 presents the demographic and clinical features that were associated with CIN / SCC on cervical screening / biopsy. Significant associations were found with abnormal vaginal bleeding, which had a positive predictive value of 7.81%. Specifically, patients with CIN or SCC were more likely to experience abnormal vaginal bleeding (*p*-value < 0.0001) and to be unmarried (p –value 0.0079). No significant associations were found for obstetrical history, patients’ region of residence, or age.

**Table 3:** Association of patient characteristics with cervical cancer screening results. Significant *p*-values are italicized

	All (*n* = 644)	Normal (*n* = 623)	CIN/SCC (*n* = 21)	Parameter Estimate / *p*-value
Age	n	Mean	SD	−0.012, 0.5198*
	644	38.78	10.90	
Marital Status	n	n (%)	n (%)	*0.0079* **
Not Married	143	133 (93.01)	10 (6.99)	
Married	501	490 (97.8)	11 (2.2)	
Region of Residence	n	n (%)	n (%)	0.3284**
Outside Goma	204	199 (97.55)	5 (2.45)	
Keshero and Himbi	166	158 (95.18)	8 (4.82)	
Mabanga and Virunga	164	161 (98.17)	3 (1.83)	
Birere	65	61 (93.85)	4 (6.15)	
Ville	44	43 (97.73)	1 (2.27)	
Obstetrical History	n	n (%)	n (%)	0.7108**
> 1 pregnancy	582	562 (96.56)	20 (3.44)	
No prior pregnancies	61	60 (98.36)	1 (1.64)	
Abnormal Vaginal Bleeding	n	n (%)	n (%)	*< 0.0001* ^+^
No	388	387 (99.74)	1 (0.26)	
Yes	256	236 (92.19)	20 (7.81)	

Keshero, Himbi, Mabanga, Virunga, Birere, and Ville are all neighborhoods in the city of Goma

SD = standard deviation

Does not account for missing data on abnormal vaginal bleeding or patients’ region of residence

* Logistic regression

**Chi-squared test

^+^Fisher exact test

## Discussion

To the best of our knowledge, this is the first published report of mass cervical cancer screening in the conflict-affected North Kivu province of the DRC. In total, 644 patients were screened between two campaigns. Since HEAL Africa Hospital had two colposcopes available, these were used to improve diagnostic accuracy over a see-and-treat approach. The hospital itself was well-staffed at the time of the screenings and the physicians and nurses who conducted the screening did so within their regular roles at the hospital such that additional resources were not required. The visiting physician volunteered her time and the colposcopes were donated. The highest associated cost was for the histopathologic examinations, which were paid for by HEAL Africa Hospital. Although the screening campaign weeks were busy for the hospital, all other patient care activities continued without any recognized adverse effects. After a half-day training on cervical cancer screening and the LEEP procedure, as well as the oversight by the expert physician during the two screening campaigns, HEAL Africa Hospital staff were able to continue with cervical cancer screening and treatment. On this basis, we believe cervical cancer screening is feasible elsewhere in DRC.

Within the screened population, the prevalence of CIN was 2.34% and the prevalence of SCC was 0.93%. The World Health Organization (WHO) recommends use of cryotherapy if the entire cervical lesion is visible, the squamocolumnar junction is visible, and the lesion does not cover more than 75% of the ectocervix, with LEEP being reserved for all other lesions. Since cryotherapy was *not* available at HEAL Africa Hospital at the time of the study, all screen positive women were treated with LEEP. Patients with CIN or SCC were more likely to report abnormal vaginal bleeding (*p*-value < 0.0001) with a positive predictive value for CIN/SCC of 7.81%.

Although recent efforts by the Gavi Vaccine Alliance aim to vaccinate 30 million girls in Africa against cervical cancer by 2020 [10], the HPV vaccine is not available in DRC at this time. While the current study did not inquire about knowledge or acceptance of the HPV vaccine, there is some existing evidence that mass HPV vaccination is feasible in lower income settings through schools and health clinics [11]. When the HPV vaccine does become available in the DRC, vaccination could be promoted through radio, church, billboard and school campaigns. DRC is among the countries without any structured or routine screening for cervical cancer. In a recent report facilitated by the World Health Organization, barriers to widespread cervical cancer care in the DRC included lack of governance and leadership, insufficient human resources within the health sector, lack of funding, unreliable supply chains for medications and equipment, and lack of awareness at the community level about the risks of cervical cancer [12]. This pilot demonstrates, however, that it is feasible to implement the “screen and treat” approach in a setting that lacks infrastructure due to prolonged armed conflict and insecurity. Patient recruitment through radio and church campaigns was successful in raising awareness and women were willing to undergo screening, and treatment where indicated. Although it was possible to perform VIA and VILI colposcopy on a large number of patients at HEAL Africa Hospital, all biopsies had to be sent to Kampala, Uganda for histopathology examination, which added to the cost. At the time of this writing, HEAL Africa Hospital is now equipped with its own laboratory that is staffed by a trained pathologist and so histopathology can be performed on site.

The single-visit “screen and treat strategy” described here has been used in other low resource countries to reduce the burden of cervical cancer [13]. VIA / VILI has been found to be more cost effective than cytology or HPV testing in areas of extreme poverty [14], with VILI reportedly having a higher sensitivity than VIA [15]. Similar to our findings, providers in other African settings have also reported that screen-and-treat methods were safe, acceptable, and feasible and that it reduced loss-to-follow up after a positive screening test [5]. Importantly, a study in Mali also determined that visual screening and treatment was sustainable in low-income settings through the maintenance of point-of-care clinics [16]. Community-based participatory interventions to improve screening for cervical [17, 18] and colon [19] cancer have also been described among underserved populations in the western context and have met with some success.

Our confirmed prevalences of CIN and SCC were lower than *suspected* but unconfirmed diagnoses at the Kayembe Hospital in Mbuji-Mayi, DRC where researchers reported that 38% of 229 patients had *suspected* CIN based on positive VIA / VILI screening and six patients (7%) had *suspected* but unconfirmed invasive cancer [20]. Our CIN prevalence of 2.34% was more consistent with results from a Kinshasa study, which revealed prevalences of CIN 1, 2 and 3 of 4.5, 1.3 and 4% respectively [21]. Within the current study, patients with CIN or SCC were more likely to report a history of abnormal vaginal bleeding (*p*-value < 0.0001), which is consistent with other studies [9, 22]. Abnormal vaginal bleeding was also frequently reported by patients with non-CIN / non-SCC cervicitis. Cervicitis is common in sexually active women and may be related to sexually transmitted infections (STI) [23]. However, STI testing was not included in the screening and additional investigation is needed to delineate the non-neoplastic causes of abnormal bleeding and cervicitis in our patient population.

Since the two mass cervical cancer screenings in 2013 and 2015, staff at HEAL Africa Hospital have noted increased numbers of women presenting for routine cervical cancer screening. It is possible that the two recruitment campaigns had the additional benefit of increasing awareness about the disease in the community. A review on barriers to cervical cancer screening in sub-Saharan Africa identified low level of awareness about services as one reason for lower uptake of screening [24]. We are not aware of any studies in eastern DRC that have documented knowledge, attitudes and practices regarding cervical cancer but one study in Kinshasa, DRC’s capital city in the western region of the country, reported a low level of knowledge, attitude and practice on cervical cancer [25]. Furthermore, a positive experience among those who attended the mass screenings (including minimal complications and no cost to the patient) may have led to the originally screened patients recommending cervical cancer screening to their family and friends. A study looking at motivations for and experiences of cervical cancer screening among HIV-positive women in Zambia found that confidential communication and support of care providers was critical to the success of cervical cancer screening programs [26]. Future research to identify motivations for cervical cancer screening amongst women in North Kivu Province would be helpful to increase uptake in the community.

This study has a number of limitations. First, since recruitment was conducted via radio campaigns and announcements in churches, women who did not listen to the radio or attend church may not have been informed. However, church attendance in DRC is very high and both Catholic and Protestant churches in and around Goma were included. Therefore, we believe that these two avenues represent some of the most effective ways to disseminate messages at the community level in North Kivu Province. Second, although it was feasible to implement mass screening at HEAL Africa Hospital, scale up of mass cervical cancer screening will likely be difficult in smaller, more remote areas where human resources are often limited and where health care providers are less likely to be trained in colposcopy and / or LEEP. However, a study from India found that a cervical cancer see-and-treat protocol performed by nurses in a resource-limited setting was deemed acceptable, safe and effective [27] and a recent critical literature review concluded that VIA and VILI can effectively be performed by nurses, midwives, and paramedic staff after a short competency-based training program [28]. Furthermore, evidence exists for the use of simple low-cost, handheld Magnivisualizers for the detection of cervical pre-cancerous and cancerous lesions where colposcopic facilities are not available [29]. Such protocols should be considered to improve access to cervical cancer screening in DRC particularly in more remote areas. Additionally, cervical pre-cancer and cancer treatment would likely be limited in smaller health care facilities without access to LEEP or cryotherapy and in centers with unreliable electricity or access to a power source. However, most hospitals in the province have access to either solar energy or generators and referral to a larger health facility could be arranged. Third, a selection bias might exist in that women with abnormal vaginal bleeding may have been more likely to present for screening and this would have inflated the reported prevalences of CIN and SCC. Finally, this study had a relatively high rate of inconclusive diagnostic biopsies, which may have resulted from biopsy specimens being too small, from inadequate sampling of the squamocolumnar junction, or due to lack of another pathologist to give a second opinion (although we cannot confirm these factors).

A number of study strengths are also noteworthy. To our knowledge this study is the first report of mass cervical cancer screening in the conflict-affected province of North Kivu. The data presented here represent the first available on the prevalence of cervical pre-cancerous and cancerous lesions. Screening was offered free of charge to all women who presented to HEAL Africa Hospital on the designated screening days and all patients for whom treatment of CIN or SCC was indicated, consented and successfully received treatment on the same visit. This study demonstrates that cervical cancer screening was acceptable to women who came forward and the treatment was generally well received with only minor complications.

## Conclusion

This study demonstrated the successful implementation of the “screen and treat” approach for detecting and treating pre-cancerous and cancerous cervical lesions in North Kivu Province of the DRC. Given that this one-visit protocol using a combination of VIA/VILI, colposcopy and LEEP was feasible and practical, it may be useful in addressing cervical cancer elsewhere in eastern DRC. However, community awareness and mobilization to address cervical cancer will be necessary, providers will have to be properly trained in VIA/VILI, colposcopy and LEEP, referral and follow ups will have to be ensured, and equipment / supplies would have to be secured if a large-scale “screen and treat” approach is to be success in reducing cervical cancer morbidity and mortality. There is ongoing need for HPV vaccination in DRC as a primary prevention strategy for cervical cancer.

## Abbreviations

CIN: Cervical intra-epithelial neoplasia
DRC: Democratic Republic of Congo
HPV: Human papillomavirus
LEEP: Loop electrosurgical excision procedure
LMIC: Low and middle- income countries
SCC: Squamous Cell Carcinoma
SD: Standard Deviation
VIA: Visual Inspection of the cervix with Acetic acid
VILI: Visual Inspection of the cervix with Lugol iodine solution
WHO: World Health Organization
